# Clinical Management of Dyslipidemia in Infants and Toddlers

**DOI:** 10.1007/s11883-025-01305-y

**Published:** 2025-06-04

**Authors:** Jennifer C. Kelley

**Affiliations:** Department of Pediatrics, Division of Pediatric Endocrinology and Diabetes, Vanderbilt University Medical Center, Nashville, USA

**Keywords:** Infants, Toddlers, Children, Hypercholesterolemia, Hypertriglyceridemia, Dyslipidemia

## Abstract

**Purpose of Review:**

Dyslipidemia can present as early as infancy however the prevalence and long-term outcomes are unclear. There is an unmet need for guidance in the evaluation and treatment approach in these patients. This review summarizes the pathophysiology of dyslipidemia in infants and toddlers and highlights potential treatment options.

**Recent Findings:**

Secondary factors unique to this population including prematurity and reliance on intravenous nutrition play a role in the pathophysiology of dyslipidemia, though primary genetic causes are also recognized. Severe hypertriglyceridemia poses a risk of acute pancreatitis in an already vulnerable population. Persistent dyslipidemia is a concern for future premature cardiovascular disease.

**Summary:**

Management of dyslipidemia is dependent on its etiology and severity. Primary and secondary causes should be considered and addressed. Although a variety of therapeutic agents are available in older children and adults, no approved therapies exist at this age, though off-label use of medications may be considered.

## Introduction

Abnormal lipid profiles in infants and young children present unique diagnostic and treatment dilemmas with limited evidence to guide clinical practice. In conjunction with rapid physiologic changes, lipid profiles also change significantly throughout the first two years of life [1–3]. Lipoprotein levels in cord blood at birth are extremely low and a progressive rise in cholesterol levels varies based on factors including sex, gestational age, parental lipid profiles, and diet [2, 4]. While evidence for tracking of lipid profiles from childhood into adulthood is well established, the exact age in childhood at which lipid levels reach a steady-state equivalent to adult levels is unclear. Population studies demonstrate correlations in lipid levels at age 6-months to those at age 5 years and into adulthood with the strongest correlations demonstrated by age 2 years [1, 4–6]. Subsequently, current guidelines do not recommend routine screening of lipid levels in children prior to age 2 years [6, 7]. Most cases of dyslipidemia in infants and toddlers are therefore detected secondary to abnormal laboratory or exam findings or due to monitoring as part of a clinical protocol, as with intravenous lipid infusions. The prevalence of dyslipidemia in this group is unknown. Further, while the etiology for dyslipidemia is broad, and acute and chronic complications may be seen, the long-term consequence on cardiovascular health is not fully established and treatment options are limited. This review aims to provide guidance in the clinical evaluation and approach to treatment of dyslipidemia in this population.

## Classification of Dyslipidemia

Classification of cholesterol levels in children and adolescents is defined by the National Expert Panel on Cholesterol Levels in Children and the Expert Panel on Cardiovascular Health Risk Reduction in Children and divided into ages 0–9 years and 10–19 years [6, 8]. These values have also been adopted in screening cut-off guidelines by the American Heart Association and American Academy of Pediatrics (AAP) [9, 10]. Classification of borderline and high levels is based upon the 75th and 95th percentiles of lipid levels in children, respectively. This classification does not define severely elevated triglyceride (TG) levels that may better represent TG values seen in severe conditions, including primary hypertriglyceridemia (HTG) and the 2012 Endocrine Society guidelines on HTG define additional levels in order to emphasize very high levels of TG that increase risk for pancreatitis [11]. Acceptable, borderline, and high lipid levels in children age 0 to 9 years include:

Total cholesterol (TC) level:

- Normal: $\:<$170 mg/dL
- Borderline: 170 to 199 mg/dL.
- High: $\:\ge\:$200 mg/dL

Low-density lipoprotein cholesterol (LDL-C) level:

- Normal: $\:<$110 mg/dL
- Borderline: 110 to 129 mg/dL.
- High: $\:\ge\:$130 mg/dL

High-density lipoprotein cholesterol (HDL-C) level:

- Normal: $\:>45$ mg/dL
- Borderline: 40 to 45 mg/dL.
- Low: $\:<40$ mg/dL

Non-HDL-C level:

- Normal: $\:<$120 mg/dL
- Borderline: 120 to 144 mg/dL.
- High: $\:\ge\:$145 mg/dL

Triglyceride level:

- Normal: $\:<$75 mg/dL
- Borderline: 75 to 99 mg/dL.
- High: 100 to 499 mg/dL.
- Very High: 500 to 999 mg/dL.
- Severe: 1000 to 1999 mg/dL.
- Very Severe: $\:\ge\:$2000 mg/dL

### Pathophysiology of Dyslipidemia in Infants and Toddlers

The etiology of dyslipidemias can be classified into primary and secondary causes. Primary dyslipidemia is typically due to inherited mutations in single or multiple genes involved in lipoprotein pathways and metabolism. Lipid abnormalities and subsequent sequelae are often severe in primary dyslipidemia [12]. In contrast, secondary dyslipidemia is acquired and may be multifactorial. Causes of secondary dyslipidemia encompass a broad range of underlying conditions, diet, lifestyle factors, and medications. Causes associated with dyslipidemia in infants and toddlers are highlighted below and listed in Table 1.

**Table 1:** Primary and secondary causes of dyslipidemia in infants and toddlers

CAUSE	LIPOPROTEIN ABNORMALITY
Primary*	
Familial chylomicronemia syndrome	↑↑ Chylomicrons
Transient infantile hypertriglyceridemia	↑-↑↑ TG
Familial hypercholesterolemia	↑-↑↑ LDL-C
Sitosterolemia	↑-↑↑ Phytosterols
**Secondary**	
Prematurity, low birth weight	↑-↑↑ TG
Intravenous lipid emulsions	↑-↑↑ TG
Renal disease	↑TC,↑ TG, ↑LDL-C, ↓HDL-C
Cholestatic liver disease	↑-↑↑ Lp-X
Hypothyroidism	↑TC,↑ TG, ↑LDL-C, ↔HDL-C
Glycogen storage disease	↑-↑↑ TG
Lipodystrophy	↑-↑↑ TG
Obesity	↑ TG, ↓HDL-C
Medications: Corticosteroids Beta-blockers Loop and thiazide diuretics Tacrolimus, sirolumus PEG-asparaginase Cyclosporine Antiretroviral therapy	Variable

*Primary conditions most likely to present at a very young age

### Primary Causes

Most primary lipid disorders are characterized by clinical presentation later in childhood/adolescence or adulthood and include conditions such as familial combined hyperlipidemia, multifactorial chylomicronemia syndrome, familial hypertriglyceridemia, and dysbetalipoproteinemia. Phenotypes in these disorders are heterogenous and frequently determined by an underlying genetic susceptibility combined with environmental and lifestyle factors such as age and weight gain. These conditions are far less likely to present in infants and toddlers. The conditions highlighted below include disorders most likely to present from birth or a very young age.

#### Familial Chylomicronemia Syndrome (FCS)

FCS is a rare monogenic condition that includes a compilation of genetic variants involved in the production and function of the lipoprotein lipase (LPL) complex. LPL is present on the luminal surface of vascular endothelial cells and functions as the rate-limiting enzyme responsible for the catabolism of triglyceride (TG)-rich lipoproteins [13, 14]. Mutations in the genes encoding the LPL complex lead to impaired lipolytic cascade and massive accumulation of chylomicrons in the plasma [15]. The estimated prevalence is 1 in 500,000 to 1,000,000 [15, 16]. TG levels may exceed 10,000 mg/dL and can present in infancy with recurrent pancreatitis [16]. Routine phlebotomy demonstrates significant lipemia. LPL activity can be estimated by measuring plasma TG levels pre- and post-intravenous heparin administration if it is determined safe to do so. Heparin solubilizes release of LPL at the vascular endothelium with subsequent decrease in plasma TG. A reduction in TG levels by 20% or more 15 min after heparin administration is considered normal, while decreases below this are consistent with impaired LPL activity [17–20]. Diagnosis with molecular genetic testing is also available, though neither of these diagnostic options are necessary to begin treatment if FCS is suspected.

#### Transient Infantile Hypertriglyceridemia (HTGTI)

HTGTI is a rare autosomal-recessive disease caused by inactivating mutations in glycerol-3-phosphate dehydrogenase 1 (GPD1) [21]. GPD1 plays an essential role in lipid metabolism within glycolysis pathways, however the pathogenesis by which mutations in GPD1 directly cause HTGTI is not clear [22, 23]. Affected individuals present in early infancy with variable hypertriglyceridemia (HTG) from 200 mg/dL to > 2,000 mg/dL, hepatosplenomegaly, elevated liver transaminases, hepatic steatosis, and failure to thrive. In most reported patients, TG levels decrease gradually by 1 year of age, though up to 70% of affected individuals may have sporadic elevation of TG into adulthood. Elevated transaminases and hepatic steatosis may also persist past infancy and can progress to cirrhosis [22, 24]. Targeted genetic testing remains the only means of diagnosis.

#### Homozygous Familial Hypercholesterolemia (HoFH)

Familial hypercholesterolemia (FH) is the most common primary lipid disorder diagnosed in children. It is most frequently caused by autosomal dominant mutations in the genes involved in the LDL receptor pathway leading to decreased clearance, and subsequent severe elevation, of LDL-C [25–27]. Heterozygous FH (HeFH) is the result of a monoallelic mutation inherited from one parent and has a prevalence of 1 in 250–500. In contrast, HoFH is caused by biallelic mutations inherited from both parents and recent estimates suggest a prevalence closer to 1 in 160,000-320,000 [28, 29]. In HeFH, LDL-C typically exceeds 160 mg/dL, whereas in HoFH, LDL-C exceeds 400 mg/dL. HeFH is typically diagnosed later in childhood following routine universal screening guidelines or after age 2 years as part of cascade screening. Unlike HeFH, HoFH often presents with overt physical exam findings in infancy and early childhood. These findings may include corneal arcus, xanthelasma, tendinous xanthomas of the Achilles tendons, patellar tendons, and extensor tendons of the hand, tuberous xanthomas frequently found along the elbows, knees and buttocks, and/or xanthomas of interdigital spaces (typically between the thumb and index finger, described as pathognomonic for HoFH). HoFH is characterized by rapid development of atherosclerotic cardiovascular disease (ACVSD) in childhood. If left untreated, severe and potentially fatal cardiovascular complications typically occur by young adulthood and premature death due to CAD progression is common by the second to third decade of life [30].

#### Sitosterolemia

Sitosterolemia is an autosomal-recessive disease caused by mutations in the genes encoding the ABC half-transporters located in the luminal surface of enterocytes and hepatocytes. These transporters limit absorption of cholesterol and plant sterols and promote fecal and biliary excretion. Transporter defects lead to excessive intestinal absorption and decreased removal of plant sterols from the liver with subsequent elevation of circulating phytosterols, which are typically reported as elevated LDL-C [31]. Sitosterolemia is frequently misdiagnosed as FH and the true prevalence is unknown. Left untreated, sitosterolemia is associated with premature ACSVD. Presentation of sitosterolemia has been reported in infants and toddlers who develop xanthomas along tendons and flexural and extensor areas of the skin. Additional findings may include macrothrombocytopenia, hemolytic anemia, splenomegaly, arthritis, and hepatic failure [32, 33]. Sitosterolemia does not respond to traditional dietary changes or therapy with statins. Diagnosis may be made through targeted genetic testing and measurement of serum plant sterol levels, which are often 30-fold higher than normal ranges.

### Secondary Causes

#### Prematurity and Low Birth Weight

Several studies have demonstrated abnormal lipid metabolism in infants with history of prematurity, small for gestational age, low birth weight, and/or intrauterine growth restriction [34, 35]. HTG is the most common lipid abnormality noted. The mechanisms involved are not fully understood, however proposed causes include immature and dysregulated LPL activity as well as decreased adipose tissue and carnitine levels leading to altered fatty acid (FA) metabolism and storage [35, 36]. Comorbidities in this population including sepsis, perinatal stress, and increased inflammatory states are also associated with HTG through cytokine-mediated increased hepatic production and release of FAs and downregulated LPL activity [37]. Birthweight and gestational age are predictive for HTG, particularly in infants receiving intravenous lipids. Weight less than 1000 g and gestational age under 28 weeks are associated with the highest risk for HTG. Holtrop et al. demonstrated that for every 100 gram decrease in birth weight, the risk for TG levels ≥200 mg/dL nearly doubled [35, 38, 39]. In most cases, the degree of HTG is mild and rarely exceeds 500 mg/dL, and resolution of HTG is typically seen with improved weight gain and enteral feeding tolerance.

#### Intravenous Lipid Emulsions (ILE)

For preterm, growth restricted, and/or critically ill infants, full nutritional support through enteral feeding is often not tolerated or is contraindicated and parenteral nutrition (PN) is necessary to meet metabolic and developmental needs [40, 41]. ILE are an essential component of PN and provide adequate calories for positive energy balance to promote growth and development, aid in the utilization of dietary protein, and deliver essential FAs [42]. However, ILE also increases the risk for HTG, particularly in vulnerable and at-risk infants. In most cases, the elevation of TG is mild, however moderate- to-severe elevation has been reported [43, 44].

#### Renal Failure and Chronic Kidney Disease (CKD)

In this age group, common causes of renal disease include inherited disorders, hypoxic ischemic insults, and nephrotoxic injury. Additionally, premature infants are prone to impaired renal function in part due to decreased nephron mass related to immature kidney development along with increased risk for neonatal acute kidney injury [45]. Dyslipidemia is a common finding in renal disease and the etiology is multifactorial [46]. In the Chronic Kidney Disease in Children Study, 45% of children with CKD were noted to have dyslipidemia, and of those, 45% had two or more lipid abnormalities [47, 48].

#### Cholestatic Liver Disease

Extra- and intrahepatic cholestasis, both acquired and inherited, may be associated with markedly abnormal lipid panels. Dyslipidemia in cholestasis is defined by the presence of lipoprotein X (Lp-X), a biliary lipoprotein complex characterized by a predominance of albumin and absence of apolipoprotein B (apoB) [49]. Lp-X shares the same density as LDL-C and standard methods to assess lipids will falsely report Lp-X as LDL-C, with levels occasionally approaching those seen in HoFH. Lp-X does not respond to traditional lipid-lowering therapies used for LDL-C. Lp-X does not appear to have an atherogenic effect, however it may be associated with development of xanthomas, hyperviscosity, thrombotic events, and laboratory abnormalities including pseudo-hyponatremia and artifactual hyperproteinemia [49, 50].

#### Hypothyroidism

Unrecognized or undertreated hypothyroidism is a well described cause of dyslipidemia [51]. Thyroid hormones play a broad role in lipoprotein metabolism and persistent hypothyroidism has been associated with increased TC and LDL-C levels, variable effects on HDL-C, and increased hepatic accumulation of TG [52, 53].

#### Inborn Errors of Metabolism

Inherited disorders of carbohydrate and adipose metabolism may present with dyslipidemia, typically characterized by HTG, in infancy and include glycogen storage diseases (GSDs) and lipodystrophies. In hepatic GSDs, particularly GSD Type 1, significant HTG is often noted along with hepatomegaly, fasting intolerance and hypoglycemia, lactic acidosis, and failure to thrive [54]. Lipodystrophies encompass a rare, heterogenous group of diseases associated with either complete or partial paucity of adipose tissue and include inherited and acquired forms. Acquired causes of lipodystrophy include exposure to certain medications, autoimmune conditions, and infections and the time interval to development of HTG varies and cannot be predicted. While many of the clinical manifestations of inherited partial lipodystrophy do not present until adolescence or later, and are dependent upon the residual amount of remaining adipose tissue, inherited complete, or generalized, forms of lipodystrophy have been associated with severe HTG and recurrent acute pancreatitis in infants [55–57].

#### Obesity

Atherogenic lipid profiles have been described in patients as young as 3 years secondary to obesity. Population-based studies have also demonstrated higher rates of HTG and low HDL-C levels in obese children < 10 years of age [58, 59]. As a result, current clinical practice guidelines from the AAP suggest that lipid evaluation in obese children age 2 years and above be considered [10].

#### Medications

Numerous medication and medication classes have been associated with the development of lipid abnormalities, and broad effects including elevation in LDL-C and TG and lowering of HDL-C are described [60, 61]. A list of these medications can be found in Table 1.

## Complications of Dyslipidemia in Infants and Toddlers

Persistent HTG in childhood and adolescence may be correlated with increased risk for future premature cardiovascular disease (CVD), though the exact association and extent to which HTG contributes to ASCVD is unclear [11, 62]. In infants and toddlers with HTG, the relationship with future CVD is even less well defined. Sustained severe elevation in LDL-C from a young age is more clearly and directly associated with increased premature CVD risk, though less likely to be identified in this age group with the exception of HoFH [63–66].

Acute complications related to dyslipidemia in infants and toddlers are typically secondary to severe HTG, typically with levels ≥ 1,000 mg/dL, and pancreatitis is the primary concerning outcome. The exact mechanism by which HTG causes pancreatitis is not understood but is thought to be related to increased plasma viscosity, impaired circulation, and ischemia with a subsequent inflammatory response in pancreatic tissue [67]. Pancreatitis is a medical emergency, and mortality may be as high as 20–30% in the general population [68, 69]. In children with FCS, recurrent spontaneous pancreatitis is common and may lead to complications including pancreatic calcification, pancreatic insufficiency, failure to thrive, and diabetes.

In premature and low weight infants with secondary HTG with levels ≥ 500 mg/dL, reported complications besides risk for pancreatitis include hyperglycemia, immune dysfunction, pneumonitis, and decreased respiratory function. Additionally, excess FA can displace circulating bilirubin from albumin, leading to unconjugated hyperbilirubinemia and risk for bilirubin-induced encephalopathy.

## Evaluation and Diagnosis

### Measurement of Lipid Levels

Detection of dyslipidemia in infants and toddlers is often dependent on the presence of specific exam findings and/or clinical scenarios. The first indication may be a lipemic blood sample noted incidentally following unrelated laboratory draws. Exam findings that may prompt strong suspicion for a lipid disorder include eruptive xanthomas and lipemia retinalis in severe HTG and tuberous and tendinous xanthomas, xanthelasma, and arcus cornealis in HoFH. Xanthomas have also been described in sitosterolemia and in the presence of Lp-X. A summary of findings that may prompt screening is available in Table 2.

**Table 2:** Screening and diagnostic considerations in the evaluation of dyslipidemia in infants and toddlers

FINDINGS THAT MAY PROMPT LIPID SCREENING IN INFANTS AND TODDLERS	
Exam Findings	- Exam findings may be normal or non-focal, however the following findings should prompt timely lipid evaluation: • Tuberous and/or tendinous xanthomas • Eruptive xanthomas • Xanthelasma • Arcus cornealis • Lipemia retinalis • Pancreatitis
Laboratory Findings	- Lipemic blood sample - Unexplained hyponatremia
Secondary Conditions	- Chronic kidney disease - Cholestatic liver disease - Obesity - Inborn errors of metabolism including lipodystrophy, glycogen storage disease
Nutrition	- Prolonged use of parenteral nutrition/intravenous lipid emulsion
**DIAGNOSTIC CONSIDERATIONS IN INFANTS AND TODDLERS**	
Lipid Panel	-When safe and tolerated: 8-hr fasting panel preferred - If fasting unsafe or not tolerated: pre-feed panel or random sample - In the setting of severe HTG, obtain a direct LDL-C
Additional Laboratory Evaluation	- Testing for secondary causes: • Thyroid function tests • Comprehensive metabolic panel • Urinalysis - With cholestatic liver disease: • Lipoprotein X • Apolipoprotein B - With marked elevation of LDL-C and systemic xanthomas: • Serum phytosterols - With severe HTG: • Lipase
Medications and Nutrition	- Evaluate recent medications for agents associated with hyperlipidemia - Consider nutrition status including continuous feeding and use of continuous dextrose infusions
Associated Conditions	- The following conditions may increase risk for dyslipidemia: • Prematurity • Sepsis and infection • Inflammatory process • Renal injury or dysfunction
Family History	- When possible, obtain parents’ lipid levels - Assess family history: • Early cardiovascular disease • Liver disease • Pancreatitis
Genetic Testing	- Consider focused testing in cases of severe elevation in LDL-C, presence of xanthomas, and sustained severe HTG - Initiate cascade screening of family members if genetic condition identified
Imaging	- With severe HTG: • Abdominal ultrasound to evaluate for pancreatitis - Severely elevated LDL-C, concern for homozygous familial hypercholesterolemia or sitosterolemia: • Electrocardiogram, echocardiogram • Arterial imaging: Cardiac CT angiography, cardiac MRI

LDL-C: Low-density lipoprotein cholesterol; TG: Triglycerides; HTG: Hypertriglyceridemia

There is no universally agreed upon protocol for monitoring of TG levels in infants receiving ILE. Practices in different institutions include TG measurement at each interval dose increase of ILE while others monitor levels only in the setting of high-risk comorbidities, such as sepsis [35]. Timing of TG measurement during ILE infusion vs. measurement during a “trough” when ILE are held also vary, though a retrospective study by Bader et al., reported no significant difference in TG levels measured during ILE infusion or during a fasting period. They concluded that serum TG measurements can be performed as part of routine laboratory testing without disruption of ILE [35, 70].

#### Diagnostic Assessment

Secondary causes of dyslipidemia should be considered in the initial assessment and include evaluation of nutritional intake, recent medication use, infection/inflammatory status, and laboratory assessment of liver, kidney, and thyroid function. In the setting of cholestasis in which LDL-C appears to be severely elevated, further assessment for the presence of Lp-X may be performed through lipoprotein electrophoresis or measurement of apoB levels.

The risk of pancreatitis increases with HTG ≥1000 mg/dL, however signs and symptoms in this age group may be nonspecific. Further laboratory assessment and imaging is prudent and include measurement of serum lipase and abdominal ultrasound. Of note, markedly elevated TG levels may interfere with serum amylase assays and result in spuriously normal results and reliance on lipase levels alone may be necessary [71].

In cases where there are no readily identifiable secondary causes, genetic evaluation for primary causes should be considered, particularly in persistent severe dyslipidemia. Several primary dyslipidemia genetic panels are available commercially. Obtaining a thorough family history, when able, is also important and promotes appropriate cascade screening of other at-risk family members. Autosomal recessive disorders may have no significant family history. Diagnostic considerations are additionally summarized in Table 2.

## Treatment

There are no current established guidelines in the management of dyslipidemia in infants or toddlers. In secondary dyslipidemia, management is typically directed at treatment and resolution of the underlying cause or adjustment in medications, if possible. When the inciting cause is expected to be transient, such as sepsis, mild to moderate elevation in lipid levels may be tolerated without further intervention and with monitoring for improvement. In conditions with a prolonged course or in more severe dyslipidemia, intervention may include conservative treatment, such as nutritional modifications, followed by off-label use of medications if necessary in severe cases. Apart from prevention of acute complications such as pancreatitis, goals for intervention include reduction of risk for future ASCVD and treatment approach at this age includes establishing lifelong heart-healthy nutritional and lifestyle practices.

### Nutrition and Lifestyle Intervention

AAP nutritional guidelines in this age group focus on guidance for adequate nutrition for normal growth and development and establishing lifelong healthy lifestyle practices [6, 10, 72]. Specific recommendations from the guidelines to address reduction in future CVD include:

- Encourage exclusive breastfeeding for as long as possible in infancy with delayed introduction of solid foods until at least 4 months. With solid food, promotion of self-selection of fruits and vegetables over sweets and simple carbohydrates and choosing monounsaturated fats is emphasized.
- Delay introduction of juice until after age 12 months and limit intake to no more than 4 ounces/day in toddlers.
- At the time of transition from formula or breastmilk, reduced fat milk may be initiated, with fat content based on growth, fat content of other nutrients, and individual risk for obesity and future CVD
- Complete avoidance of screen time prior to age 2 years and limiting screen time to 1–2 hours daily in toddlers. Caregivers are encouraged to model and develop an environment that promotes active lifestyles.
- In children with persistent dyslipidemia, the Cardiovascular Health Integrated Lifestyle Diet (CHILD-1) is the first stage recommendation in dietary modifications. Recommendations include limiting fat intake to 25–30% of total daily caloric intake, avoiding trans fats and sugar-containing beverages other than skim milk, and increasing fiber intake.

### TPN and ILE Adjustments

The ideal composition of ILE in neonates is not yet established. Multiple formulations are available and may exclusively use soybean or fish oil, as well as multi-oil options that include medium chain TGs, olive oil, and coconut oil [35, 73]. In infants with intestinal failure-associated liver disease (IFALD), ILE formulations composed only of fish oil (FO ILE) have been associated with lower TG concentrations, though evidence thus far does not support the preferential use of FO ILE in neonates without IFALD to prevent HTG [74, 75]. The European Society for Pediatric Gastroenterology, Hepatology and Nutrition recommends limiting serum TG levels to 265 mg/dL in infants on ILE, however this is not universally adopted [35, 76, 77]. Reduction in ILE doses in response to HTG increases the risk for development of an essential FA disorder (EFAD). In a recent review of HTG management in preterm infants on ILE by Chan et al., recommendations include minimizing excessive dextrose infusion, ensuring ILE are infused over 20 to 24 hours and avoiding inappropriately high or rapid infusions, and use of FO ILE in the setting of IFALD [35]. If ILE must be reduced or held in the setting of severe HTG, monitoring for and avoidance of a potential EFAD is essential.

### Pharmacological Therapies

Pharmacological treatment of severe dyslipidemia in very young children may be considered when dietary and lifestyle changes are ineffective. However, none of the agents described below are approved by the United States Food and Drug Administration (FDA) for use in infants or toddlers, and the safety, clinical efficacy, and cost-effectiveness is not established in this group. The use of these treatments should thus be considered only in severe cases and are best managed under the guidance of a pediatric lipidologist.

### LDL-C Predominant Dyslipidemia

- *Statins* inhibit HMG-CoA reductase, the rate-limiting enzyme in cholesterol synthesis in hepatocytes. They are the most prescribed lipid-lowering medications worldwide and are effective in the primary and secondary prevention of ASCVD, particularly in FH. They are not indicated in treating HeFH in infants and toddlers, however they are used off-label in the treatment at all ages in HoFH. Current FDA-approval of statins for children include: rosuvastatin from age 6 years; pitavastatin and pravastatin in children from 8 years; and atorvastatin, fluvastatin, lovastatin and simvastatin in children from age 10 years [78].
- *Ezetimibe* is a selective-absorption inhibitor and is often used in combination with statins for further lowering of LDL-C levels. Additionally, it is the main treatment, along with dietary adjustments, for sitosterolemia. The licensed formulation of ezetimibe is in 10 mg tablets given once daily [78]. It is FDA approved for use in children 10 years of age and older [79].
- *Plant sterols* compete with dietary cholesterol for absorption within enterocytes. They are naturally occurring in food and may also be supplemented. Their use as part of overall nutritional management of elevated LDL-C has been described in children age 2 years and older, and appear to be safe and well tolerated though their effect on long-term cardiovascular risk reduction is not fully defined [80]. Doses in children are generally 1.3 to 2.6 g daily. As a supplement, the FDA has approved a health claim for the use of stanols but does not regulate their use [81].

### TG Predominant Dyslipidemia

- *Fibric acid derivatives* reduce VLDL production and increase TG clearance through enhancement of LPL activity and are effective in treating HTG. Reported use in infants and toddlers is limited to cases of extreme HTG, typically in the setting of elevated risk for, or history of, pancreatitis. Fenofibrate is the most common option in these cases. The licensed formulation of fibrates starts at doses of 40 mg to 54mg taken once daily. Currently, there are no fibric acid derivatives with FDA approval for use in patients under age 18 years.
- *Long chain omega-3 FAs*, eicosapentaenoic acid (EPA) and docosahexaenoic acid (DHA), are thought to improve TG levels by increasing FA oxidation and decreasing FA substrate availability in the liver, with subsequent reduction in VLDL synthesis. In children, they are primarily available in fish oil (FO) or flaxseed supplements. Effective doses in adults start at 2 g of omega-3 FA daily however safe and effective doses in infants and toddlers are not established. In a recent randomized clinical trial of 70 full-term infants with IUGR, omega-3 FA initiation at a dose of 40 mg/kg/day resulted in significantly reduced TG levels by a mean of 43% compared to placebo and were generally well tolerated, suggesting a potential role for omega-3 FAs in management of HTG in infants starting at lower doses than in adults [82]. At this time, pediatric indications are not included in FDA approved formulations of omega-3 FAs.
- *Insulin* has been utilized in the treatment of persistent HTG with pancreatitis. Acute management of severe HTG with pancreatitis includes maintaining NPO status with appropriate IV hydration [16, 83]. If effective TG reduction is not achieved, the addition of a continuous infusion of regular insulin is warranted. Insulin is a rapid and potent promoter of LPL synthesis [19]. Current protocols in adults include initiation of regular insulin starting at 0.1 units/kg/hour, with hourly monitoring of blood glucose levels and titration of the infusion rate [84]. In infants and toddlers, in which there are no FDA-approved or standardized protocols available, lower initial infusion rates may be desired to avoid hypoglycemia and can be gradually advanced with appropriate glucose monitoring.

FCS is the exception to these treatments as traditional TG-lowering therapies rely on upregulating LPL activity and are ineffective due to the lack of functional LPL in this condition. While fibrates have been used in infants and toddlers with FCS, successful reduction of TG is typically limited. Additionally, omega-3 FA may exacerbate TG levels due to a potential increase in chylomicron production [85]. The only currently available treatment is strict restriction of fat intake to ≤ 10–15% of total daily caloric consumption [13]. In infants, this is accomplished by providing a formula with limited long chain TG (enough to meet essential FA requirements) and composed primarily of medium chain TG (MCT). MCTs are hydrolyzed in the intestinal mucosa to FAs and do not require lipase for absorption and thus can provide adequate additional calorie support. As solid food is added to the diet, lifelong adherence to the strict fat restriction is crucial and best supported by ongoing management with a registered dietitian.

### Non-Pharmacological Therapies

In cases of persistent severe HTG, pancreatitis or worsening clinical status, plasmapheresis may be initiated. Plasmapheresis separates blood from selective components and allows for the direct elimination of lipoprotein particles. It is also effective at reducing plasma viscosity [16, 86, 87]. However, the use of plasmapheresis in pediatric patients is limited by technical difficulty and availability and should only be performed in experienced medical centers [83, 86]. If plasmapheresis is not available or feasible, whole blood exchange transfusions have also been described for rapid clearance of TG in emergencies, including in an infant with multi-organ failure and severe HTG [83, 88].

In HoFH, patients rarely achieve optimal lowering of LDL-C with medication alone and most require the addition of lipoprotein apheresis, which selectively removes apoB-containing particles from the circulation. Apheresis must be performed every 1 to 2 weeks for effective management and requires a medical center with expertise in its use in this setting [65, 89].

### New and Developing Therapies

Novel treatments for FCS are currently being explored, though trials are limited to adult patients. Research using gene therapy are ongoing, however the first and only gene therapy, alipogene tiparvovec, was approved in the European Union but was withdrawn due to excessive cost and limited efficacy [90, 91]. A new medication, volanesorsen, is an antisense oligonucleotide that targets the mRNA of apolipoprotein C-III mRNA and has been shown to effectively reduce TG in FCS but is currently not FDA-approved and has not been studied in children [92].

There have been several recent developments in therapeutic options for HoFH. Of these, monoclonal antibodies targeting proprotein convertase subtilisin/kexin type 9 (PCSK9), evolucumab and alirocumab (approved from age 10 and 8 years, respectively), and angiopoietin-like 3 (ANGPTL3), evinacumab (approved from age 5 years), are the only additional options FDA approved in children [93–95]. In addition to new therapeutic agents, several trials targeted at gene therapy for HoFH are also ongoing [96].

## Conclusion and Future Directions

Recognition of dyslipidemia in infants and toddlers is important due to potential acute and chronic complications. However, there is a paucity of evidence to guide evaluation, management, and long-term outcomes in this population. Increased reporting of dyslipidemia, evaluation of treatment through clinical trials, and expert consensus are needed to improve our understanding and clinical approach to dyslipidemia in these patients. Optimal management of severe and persistent dyslipidemia may involve the assistance of pediatric lipid specialists, geneticists, and dietitians. Recently developed medications targeting various lipid conditions are available in adults and there are several ongoing novel treatments in development, including targeted genetic therapies, though their potential use in the pediatric population, especially infants and young children, is unclear.

### Key References

1.• Oyri LKL, Bogsrud MP, Kristiansen AL, Myhre JB, Astrup H, Retterstol K, et al. Cholesterol at ages 6, 12 and 24 months: Tracking and associations with diet and maternal cholesterol in the Infant Cholesterol Study. Atherosclerosis. 2021;326:11 − 6. doi: 10.1016/j.atherosclerosis.2021.04.017.

**Prospective cohort study tracking total cholesterol trends in 258 infants over the first 12 months of life.**

2.• Taageby Nielsen S, Mohr Lytsen R, Strandkjaer N, Juul Rasmussen I, Sillesen AS, Vogg ROB, et al. Significance of lipids, lipoproteins, and apolipoproteins during the first 14–16 months of life. Eur Heart J. 2023;44(42):4408-18. doi: 10.1093/eurheartj/ehad547.

**Prospective cohort study 258 infants tracking multiple lipid measures over the first 16 months of life.**

3.• Gidding SS, Wiegman A, Groselj U, Freiberger T, Peretti N, Dharmayat KI, et al. Paediatric familial hypercholesterolaemia screening in Europe: public policy background and recommendations. Eur J Prev Cardiol. 2022;29(18):2301-11. doi: 10.1093/eurjpc/zwac200.

**Policy recommendations for screening and recognition of familial hypercholesterolemia in pediatric patients.**

4.•• Hampl SE, Hassink SG, Skinner AC, Armstrong SC, Barlow SE, Bolling CF, et al. Clinical Practice Guideline for the Evaluation and Treatment of Children and Adolescents With Obesity. Pediatrics. 2023;151(2). doi: 10.1542/peds.2022-060640.

**Updated clinical practice guideline for the evaluation and management of pediatric obesity from the American Academy of Pediatrics**.

6.• Ziolkowska S, Kijek N, Zendran I, Szuster E, Barg E. Familial hypercholesterolemia - treatment update in children, systematic review. Pediatr Endocrinol Diabetes Metab. 2022;28(2):152 − 61. doi: 10.5114/pedm.2022.116112.

**Systematic review of current guidelines and statements on the treatment of pediatric familial hypercholesterolemia.**

7.• Chan AP, Robinson DT, Calkins KL. Hypertriglyceridemia in Preterm Infants. Neoreviews. 2022;23(8):e528-e40. doi: 10.1542/neo.23-8-e528.

**Review of the pathophysiology and approach to management of hypertriglyceridemia in preterm infants on intravenous lipid emulsions.**

8. • Elsheikh M, El Amrousy D, El-Mahdy H, Dawoud H, Harkan A, El-Barky A. Lipid profile after omega-3 supplementation in neonates with intrauterine growth retardation: a randomized controlled trial. Pediatr Res. 2023;94(4):1503-9. doi: 10.1038/s41390-023-02632-z.

**Random controlled clinical trial assessing lipid profile changes in response to omega-3 fatty acid supplementation in 70 infants with intrauterine growth failure.**

9.•• Bruckert E, Caprio S, Wiegman A, Charng MJ, Zarate-Morales CA, Baccara-Dinet MT, et al. Efficacy and Safety of Alirocumab in Children and Adolescents With Homozygous Familial Hypercholesterolemia: Phase 3, Multinational Open-Label Study. Arterioscler Thromb Vasc Biol. 2022;42(12):1447-57. doi: 10.1161/ATVBAHA.122.317793.

**Open label Phase 3 clinical trial of alirocumab in 18 pediatric patients with homozygous familial hypercholesterolemia.**

10. •• Raal FJ, Hegele RA, Ruzza A, Lopez JAG, Bhatia AK, Wu J, et al. Evolocumab Treatment in Pediatric Patients With Homozygous Familial Hypercholesterolemia: Pooled Data From Three Open-Label Studies. Arterioscler Thromb Vasc Biol. 2024;44(5):1156-64. doi: 10.1161/ATVBAHA.123.320268.

**Pooled dated from three open label Phase 3 clinical trials of elirocumab in 39 pediatric patients with homozygous familial hypercholesterolemia.**

11. •• Wiegman A, Greber-Platzer S, Ali S, Reijman MD, Brinton EA, Charng MJ, et al. Evinacumab for Pediatric Patients With Homozygous Familial Hypercholesterolemia. Circulation. 2024;149(5):343 − 53. doi: 10.1161/CIRCULATIONAHA.123.065529.

**Open label Phase 3 clinical trial of evinacumab in 14 pediatric patients with homozygous familial hypercholesterolemia.**

## Data Availability

No datasets were generated or analysed during the current study.
